# BOLD Noise Assumptions in fMRI

**DOI:** 10.1155/IJBI/2006/12014

**Published:** 2006-07-30

**Authors:** Alle Meije Wink, Jos B. T. M. Roerdink

**Affiliations:** ^1^ Brain Mapping Unit, Department of Psychiatry, University of Cambridge, Addenbrooke's Hospital, Hills Road, Cambridge CB2 2QQ, United Kingdom; ^2^ Institute for Mathematics and Computing Science, University of Groningen, P.O. Box 800, Groningen 9700 AV, The Netherlands; ^3^ Institute for Behavioral and Cognitive Neurosciences and BCN Neuroimaging Center, University of Groningen, , The Netherlands

## Abstract

This paper discusses the assumption of Gaussian noise in the blood-oxygenation-dependent (BOLD) contrast for functional MRI (fMRI). In principle, magnitudes in MRI images follow a Rice distribution. We start by reviewing differences between Rician and Gaussian noise. An analytic expression is derived for the null (resting-state) distribution of the difference between two Rician distributed images. This distribution is shown to be symmetric, and an exact expression for its standard deviation is derived. This distribution can be well approximated by a Gaussian, with very high precision for high SNR, and high precision for lower SNR. Tests on simulated and real MR images show that subtracting the time-series mean in fMRI yields asymmetrically distributed temporal noise. Subtracting a resting-state time series from the first results in symmetric and nearly Gaussian noise. This has important consequences for fMRI analyses using standard statistical tests.


Hindawi Publishing Corporation
International Journal of Biomedical Imaging
Volume 2006, Article ID 12014, Pages 1–11
DOI 10.1155/IJBI/2006/12014

### BOLD Noise Assumptions in fMRI

**Alle Meije Wink[1] and Jos B. T. M. Roerdink[2, 3]**

[1] *Brain Mapping Unit, Department of Psychiatry, University of Cambridge, Addenbrooke's Hospital, Hills Road, Cambridge CB2 2QQ, UK*
[2] *Institute for Mathematics and Computing Science, University of Groningen, P.O. Box 800, 9700 AV Groningen, The Netherlands*
[3] *Institute for Behavioral and Cognitive Neurosciences and BCN Neuroimaging Center, University of Groningen, The Netherlands*

Received 30 January 2006; Revised 13 June 2006; Accepted 17 June 2006

This paper discusses the assumption of Gaussian noise in the blood-oxygenation-dependent (BOLD) contrast for functional MRI (fMRI). In principle, magnitudes in MRI images follow a Rice distribution. We start by reviewing differences between Rician and Gaussian noise. An analytic expression is derived for the null (resting-state) distribution of the difference between two Rician distributed images. This distribution is shown to be symmetric, and an exact expression for its standard deviation is derived. This distribution can be well approximated by a Gaussian, with very high precision for high SNR, and high precision for lower SNR. Tests on simulated and real MR images show that subtracting the time-series mean in fMRI yields asymmetrically distributed temporal noise. Subtracting a resting-state time series from the first results in symmetric and nearly Gaussian noise. This has important consequences for fMRI analyses using standard statistical tests.

Copyright © 2006 A. M. Wink and J. B. T. M. Roerdink. This is an open access article distributed under the Creative Commons Attribution License, which permits unrestricted use, distribution, and reproduction in any medium, provided the original work is properly cited.

### 1. INTRODUCTION

Functional magnetic resonance imaging (fMRI) measures activity in different areas of the brain under different experimental conditions (e.g., active, rest). In the medical imaging literature, magnitudes in MR images are assumed to follow a Rice distribution [1–7], first studied by Rice [8, pages 100–103]. Most statistical analyses of fMRI images test the difference between experimental conditions against a *null distribution*, which applies when no task is performed. Parametric statistical fMRI analysis often assumes Gaussian noise [9–14], but findings contradicting this assumption have already been reported by Hanson and Bly [15], and tests for the distribution of the residual (noise) signal in fMRI data sets have been developed [16].

In this paper, we examine the properties of Rician noise, and the distribution of resting state images that are made by pairwise subtraction of MR images. Most standard tests, such as the $t$-test, $F$-test, and the $z$-test, rely on Gaussian distributed noise. Petersson et al. [17] argue that with Gaussian spatial smoothing, many degrees of freedom, and the multivariate central limit theorem, these tests are valid in functional neuroimaging, but they warn that low-count PET data show departures from normality. Similar effects can be seen in functional MR images. The Rician probability density function is very asymmetric if the signal is weak compared to the noise, so for low signal intensities and with a low signal-to-noise ratio (SNR), Rician noise and Gaussian noise behave very differently and the Rician distribution has to be taken into account in order to prevent biased statistical results.

This problem is important for fMRI, because the scans may have relatively low SNRs, and the values of the BOLD contrast are very small compared to the noise. This is especially true for data with high temporal and/or spatial resolution: this will inevitably lead to lower SNR values.

The remainder of this paper is organised as follows. Section 2 introduces the Rician noise model for MR images. Section 3 derives analytical expressions for the probability distribution of the difference between two MR images, which are verified in a series of tests on synthetic noise images. Section 4 investigates the noise distributions in MR template images contaminated with noise and in a real fMRI time series, and discusses implications for the design of fMRI experiments. Section 5 contains some general conclusions.

### 2. NOISE IN MR IMAGES

During image acquisition in an MR scanner, magnetic fields are transmitted in pulses varying in frequency and phase. Voxel locations are selected by frequency and phase, and the resulting data consist of complex values. The frequency space in which these data are represented is known as the $k$-space. The values in the real and imaginary parts of the image are Gaussian distributed. The $k$-space data are transformed to a Cartesian space via an inverse Fourier transform (IFT). The noise distribution in the resulting image is still Gaussian, because the IFT is a linear transform.

Most applications of MR imaging only use the magnitudes of the signal, because those magnitudes represent a physical property of the scanned object [18]. Let $A(\mathbf{x})$ represent the magnitude of the MR image at voxel location $\mathbf{x}$ in the absence of noise. The magnitude $r(\mathbf{x})$ of the signal at voxel location $\mathbf{x}$ in the magnitude image is

$$r(\mathbf{x}) = \sqrt{\left(A(\mathbf{x}) + n_1(\mathbf{x})\right)^2 + n_2(\mathbf{x})^2}, \quad n_1(\mathbf{x}), n_2(\mathbf{x}) \sim N(0, \sigma^2), \tag{1}$$

where $n_1(\mathbf{x})$ and $n_2(\mathbf{x})$ are the real and imaginary parts of the noise and $N(0,\sigma^2)$ is the Gaussian distribution with mean zero and standard deviation $\sigma$.

The magnitude signal in each voxel $\mathbf{x}$ is Rician distributed [1–3], that is, $\text{Prob}[r \le r(\mathbf{x}) \le r + \mathrm{d}r] = p_{A(\mathbf{x}),\sigma}(r)$, where $p_{A,\sigma}(r)$ is the Rician probability density with parameters $A$ and $\sigma$ defined by

$$p_{A,\sigma}(r) = \begin{cases} 0, & r < 0 \\ \dfrac{r}{\sigma^2} e^{-(A^2+r^2)/2\sigma^2} I_0\left(\dfrac{Ar}{\sigma^2}\right), & r \ge 0, \end{cases} \tag{2}$$

where

$$I_k(z) = \frac{1}{\pi} \int_0^{\pi} e^{z\cos(\theta)} \cos(k\theta)\mathrm{d}\theta \tag{3}$$

is the modified Bessel function of the first kind of order $k$, $k \in \mathbb{N}$. Figure 1 shows the Rician probability density function (pdf) for varying values of $A$ and $\sigma$. The shape of the PDF changes with both parameters. The distribution for $A = 0$ is called the *Rayleigh distribution*. For high SNRs, the Rician distribution approaches a Gaussian distribution [3].

The mean $\mu_r = \int_0^{\infty} r p_{A,\sigma}(r)\mathrm{d}r$ of the Rice distribution is given by [8, pages 100–103, Appendix 4B]

$$\mu_r = \sigma\sqrt{\frac{\pi}{2}} e^{(-z^2/4)4} \left\{ \left[1 + \frac{z^2}{2}\right] I_0\left(\frac{z^2}{4}\right) + \frac{z^2}{2} I_1\left(\frac{z^2}{4}\right) \right\}, \tag{4}$$

where $z = A/\sigma$ is the SNR, and $I_k$, $k = 0, 1$, is defined in (3). The standard deviation $\sigma_r = \sqrt{\int_0^{\infty} r^2 p_{A,\sigma}(r)\mathrm{d}r - \mu_r^2}$ of the Rice distribution satisfies the relation [3]

$$\sigma_r = \sqrt{A^2 + 2\sigma^2 - \mu_r^2}. \tag{5}$$

As $A/\sigma$ goes to infinity, these formulas yield $\mu_r \to A$, $\sigma_r \to \sigma$, that is, the mean approaches the noise-free intensity and the standard deviation approaches the corresponding value of the underlying noise distribution $N(0, \sigma^2)$.

MR noise was modelled by computing the intensity distribution as in (1). To make a Rice-distributed noisy image from a real-valued noise-free image $f(\mathbf{x})$, we use the

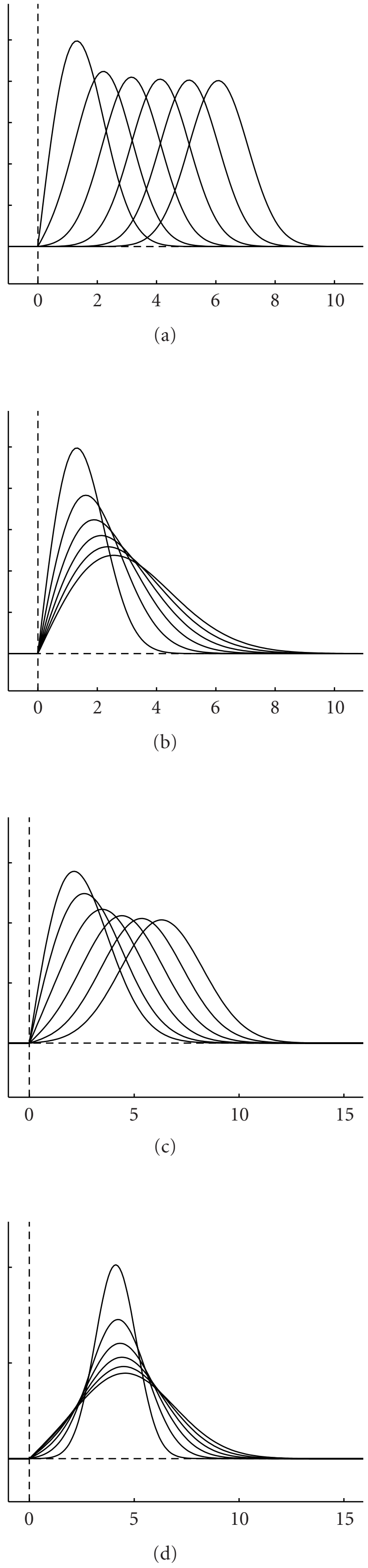

Figure 1: (a) Rician PDFs for $\sigma^2 = 1$ and $A \in \{1, \ldots, 6\}$, (b) Rician PDFs for $A = 1$ and $\sigma_r^2 \in \{1, \ldots, 6\}$, (c) Rician PDFs for $\sigma^2 = 4$ and $A \in \{1, \ldots, 6\}$, (d) Rician PDFs for $A = 4$ and $\sigma_r^2 \in \{1, \ldots, 6\}$.

following procedure for each voxel location $\mathbf{x}$:

(1) $n_1(\mathbf{x}), n_2(\mathbf{x}) \sim N(0, \sigma^2)$,
(2) $r(\mathbf{x}) = \sqrt{[f(\mathbf{x}) + n_1(\mathbf{x})]^2 + n_2(\mathbf{x})^2}$.

Again, the noisy image is denoted by $r(\mathbf{x})$. The local SNR is controlled through the ratio $f(\mathbf{x})/\sigma$, where $f(\mathbf{x})$ and $\sigma$ determine $\mu_r$ and $\sigma_r$ as described in (4) and (5), respectively.

### 3. MATHEMATICAL ANALYSIS OF fMRI NOISE

### 3.1. Statistical testing in fMRI

In fMRI analysis, one searches for activation in certain brain areas via statistical hypothesis testing. The null hypothesis $H_0$ states that there is no activation, other hypotheses correspond to several kinds of activation. Most fMRI analysis methods, such as statistical parametric mapping [13], use the general linear model (GLM). The GLM treats fMRI responses as the outputs of a linear time-invariant (LTI) system using a number of temporal basis functions, $f_1(\cdot), \ldots, f_M(\cdot)$, called *explanatory variables*. The GLM has the form

$$Y_{k,s} = \beta_{k,1} f_1(t_s) + \cdots + \beta_{k,M} f_M(t_s) + e_{k,s}, \quad (6)$$

where $Y_{k,s}$ is the observed data at voxel $k$, $k = 1, \ldots, N$, and time index $s$, $s = 1, \ldots, T$; $f_m(t_s)$ is value of the $m$th basis function at time $t_s$, $m = 1, \ldots, M$; the $\beta_{k,m}$ are weight factors of each temporal component at each voxel (to be determined from the measurements); and $e_{k,s}$ is the error (noise) at voxel $k$ and time index $s$. In matrix form, the GLM may be succinctly written as

$$\mathbf{Y} = \mathbf{X}\boldsymbol{\beta} + \mathbf{e}, \quad (7)$$

where $\mathbf{Y}$ is a $T \times N$ matrix, $\mathbf{X}$ is the $T \times M$ design matrix containing the $f_m(t_s)$ values, $\boldsymbol{\beta}$ is $T \times N$ weight matrix, and $\mathbf{e}$ is the $T \times N$ residual matrix containing the part of the signal not modelled by any component in $\mathbf{X}$. Statistical parametric tests often assume that the error values in $\mathbf{e}$ are independent and identically normally distributed, that is, $e_{k,s} \sim N(0, \sigma_k^2)$, where the standard deviation may depend on the voxel location.

In brain activation studies, one considers an equation of the form (6) for both the activated and the rest (null) condition. Let $Y_{k,s}^q$ denote the observed signals under condition $q$ (0 = "rest," 1 = "active"). Then a test statistic is formed at each voxel, for example, a t-statistic $T_k$ defined by

$$T_k = \frac{\overline{Y}_k^1 - \overline{Y}_k^0}{S_k^2 \cdot (2/M)}, \quad (8)$$

where $\overline{Y}_k^q$ is the temporal average per voxel and $S_k^2$ is the pooled variance estimate, that is,

$$\overline{Y}_k^q = \frac{1}{T} \sum_{s=1}^{T} Y_{k,s}^q,$$
$$S_k^2 = \frac{1}{2M-2} \sum_{q=0}^{1} \sum_{s=1}^{T} \left(Y_{k,s}^q - \overline{Y}_k^q\right)^2. \quad (9)$$

Under the assumption of Gaussian noise, that is, $e_{k,s}^q \sim N(0, \sigma_k^2)$, $Y_{k,s}^q$ is normally distributed with mean $\mu_q$ and standard deviation $\sigma_k$, and also the differences $Y_{k,s}^q - \overline{Y}_k^q$ are normally distributed. This implies that $T_k \sim t_{2M-2}$, that is, $T_k$ has a $t$-distribution with $2M - 2$ degrees of freedom *under the null hypothesis* $H_0 : \mu_0 = \mu_1$, that is, no mean effect of activation occurs. For this reason the distribution of $T_k$ under this hypothesis is called the *null distribution*. Voxels where this hypothesis can be rejected are therefore designated as activated voxels. The significance of a certain observed voxel value is expressed by a so-called *p-value*, which is the probability of that voxel's intensity being attributable to mere chance. A $p$-value is calculated as the area under the graph of the $t$-distribution to the right of a given intensity value on the horizontal axis. A low $p$-value (say lower than 0.05) indicates that the measured value is probably not due to mere chance, that is, that it is a real activation.

The Rician distribution which applies to MRI data has a heavier right tail than a Gaussian, so $p$-values based on a Gaussian noise assumption with the standard deviation of the Rice distribution will be too low, introducing false positives. Hanson and Bly [15] found similar deviations, using gamma distributions instead of Rician PDFs.

As we have seen, it is the *null distribution* which is needed to compute $p$-values. From the discussion above, it is apparent that a sufficient condition for the usual statistical analysis to hold is that the *difference signal* at each voxel corresponding to the case of no activation has a Gaussian distribution. Therefore, our object of study in the remainder of this paper is the distribution of the noise in difference images of Rician-distributed MRI images without activation. As we will see, this distribution is indeed very close to a Gaussian, albeit with a standard deviation different from that of the initial Rice distribution.

### 3.2. Null distribution of the difference fMRI signal

The difference of two noisy versions $r_1(\mathbf{x})$ and $r_2(\mathbf{x})$, containing Rician noise, of the same underlying image $f(\mathbf{x})$, is *not* Rician distributed. Let the null image $s(\mathbf{x})$ be defined as $s(\mathbf{x}) = r_2(\mathbf{x}) - r_1(\mathbf{x})$. Its probability density function (PDF) is denoted by $C_{A,\sigma}(s)$, where we write $A$ instead of $f(\mathbf{x})$ and $\sigma$ is the standard deviation of the underlying noise distribution (cf. (1)). Then $C_{A,\sigma}(s)$ is the probability that the value of the difference $s(\mathbf{x})$ falls in an infinitesimal interval around $s : C_{A,\sigma}(s) = \text{Prob}[s \le s(\mathbf{x}) \le s + \text{d}s]$. We will refer to $C_{A,\sigma}(s)$ as the null distribution.

Since it is easy to see that $C_{A,\sigma}(s)$ is symmetric, that is, $C_{A,\sigma}(s) = C_{A,\sigma}(-s)$, we have the following expression valid for arbitrary values of $s \in \mathbb{R}$:

$$\begin{aligned} C_{A,\sigma}(s) &= \iint_0^\infty p_{A,\sigma}(r_1) p_{A,\sigma}(r_2) \delta(r_2 - r_1 - |s|) \text{d}r_1 \text{d}r_2 \\ &= \int_0^\infty p_{A,\sigma}(r_1) p_{A,\sigma}(r_1 + |s|) \text{d}r_1, \end{aligned} \quad (10)$$

where $\delta(r)$ denotes the Dirac delta function. That is, $C_{A,\sigma}(s)$ is the cross-correlation of two identical Rice distributions.

The mean $\mu_s$ and standard deviation $\sigma_s$ of the null distribution $C_{A,\sigma}(s)$ are given by

$$\mu_s = 0, \qquad \sigma_s = \sqrt{2}\sigma_r, \tag{11}$$

where $\sigma_r$ is the standard deviation of the Rice distribution, see (5). For the derivation, see Appendix A.1. In the case $A = 0$, the PDF of $r_1$, as well as that of $r_2$, is

$$p_{0,\sigma}(r) = \frac{r}{\sigma^2} e^{-r^2/2\sigma^2}. \tag{12}$$

For the Rayleigh case ($A = 0$), the integral in (10) can be explicitly evaluated. The resulting expression for $C_{0,\sigma}(s)$ is

$$\frac{1}{2\sigma} e^{-s^2/4\sigma^2} \left[ \frac{|s|}{2\sigma} e^{-s^2/4\sigma^2} + \frac{\sqrt{\pi}}{2} \left( 1 - \frac{s^2}{2\sigma^2} \right) \operatorname{erfc}\left( \frac{|s|}{2\sigma} \right) \right], \tag{13}$$

where $\operatorname{erfc}(z) = (2/\sqrt{\pi}) \int_z^\infty e^{-t^2} \mathrm{d}t$ is the complementary error function [19]. For the derivation of this formula, we refer to Appendix A.2.

The following experiments investigate how well the PDF $C_{A,\sigma}(s)$ can be approximated by a Gaussian distribution.

### 3.3. Numerical approximation by a normal distribution

The distribution $C_{A,\sigma}(s)$, see (10), was numerically approximated by a Gaussian via the Levenberg-Marquardt curve-fitting algorithm. The fit was carried out on an interval centered around zero with negligible function values outside this interval. Figure 2 shows the PDF $C_{A,\sigma}(s)$, as well as the Gaussian fitted to this distribution, for a number of values of $A$ and $\sigma$. The plots show an excellent fit.

Table 1 presents some quantitative results. It shows, for various values of $A$ and $\sigma$, (i) the exact standard deviation $\sigma_s = \sqrt{2}\sigma_r$ of the null distribution (11), where $\sigma_r$ was computed according to (4)-(5); (ii) the width $\sigma^{\text{Gauss}}$ of the Gaussian fitted to $C_{A,\sigma}(s)$; and (iii) the mean square error of the difference between $C_{A,\sigma}(s)$ itself and the fitted Gaussian. The difference between the width $\sigma^{\text{Gauss}}$ of the fitted Gaussian and the exact value $\sigma_s$ is very small, especially for high SNR (i.e., $A/\sigma$). Since $\sigma_r$ approaches $\sigma$ for high SNR (see Section 2), $\sigma_s$ approaches $\sqrt{2}\sigma$ in this limit. The mean square error should decrease when the SNR increases; this is confirmed by the experimental results. The null distribution does not have a heavy tail (it is slightly lighter than a Gaussian of width $\sigma$, see below). Function values outside the interval used in the fitting procedure represent a negligible portion of the distribution. Therefore, $p$-values from statistical tests based on Gaussian noise with an estimated standard deviation $\sigma^{\text{Gauss}}$ will be very accurate, and (because of the light tail) where there is a difference, the estimates will be conservative.

### 3.4. Tail of the null distribution

An important property of the PDF $C_{A,\sigma}(s)$ for statistical fMRI analysis is the tail behaviour (as $|s|$ approaches infinity) of the distribution under the null hypothesis, because this determines the $p$-value corresponding to a certain threshold (cf. Section 3.1).

Table 1: Accuracy of Gaussian fits to the PDF $C_{A,\sigma}$ for various values of $A$ and $\sigma$. Shown are the exact standard deviation $\sigma_s$ computed from (11), the width $\sigma^{\text{Gauss}}$ of the fitted Gaussian, and the mean square error of the difference between the exact distribution and the fitted Gaussian.

| $A$ | $\sigma$ | $\sigma_s$ | $\sigma^{\text{Gauss}}$ | Error |
|---|---|---|---|---|
| 0 | 1 | 0.9265 | 0.9103 | 0.0080 |
| 0 | 3 | 2.7795 | 2.7315 | 0.0045 |
| 0 | 5 | 4.6325 | 4.5526 | 0.0035 |
| 2 | 1 | 1.2933 | 1.3071 | 0.0030 |
| 2 | 3 | 3.0463 | 3.0085 | 0.0035 |
| 2 | 5 | 4.8079 | 4.7291 | 0.0033 |
| 8 | 1 | 1.4086 | 1.4086 | 0.0000 |
| 8 | 3 | 4.0552 | 4.0780 | 0.0008 |
| 8 | 5 | 6.1567 | 6.2188 | 0.0013 |

For the limiting cases of low and high SNR, that is, $A = 0$ and $A/\sigma$ large, we mathematically analysed the behaviour of the PDF $C_{A,\sigma}(s)$ when $|s|$ becomes very large. The details are presented in Appendix A.3. We find that both in the Rayleigh case ($A = 0$) and for large values of $A/\sigma$, the tails of the distribution (10) are lighter than the tail of a Gaussian distribution:

$$C_{A,\sigma}(s) \sim \text{constant} \cdot \frac{1}{|s|} e^{-(|s|-A)^2/2\sigma^2}, \quad s \longrightarrow \infty, \tag{14}$$

where the constant depends on $A$ and $\sigma$. This is a Gaussian tail of width $\sigma$ multiplied by a factor $1/|s|$, which means that the distribution approaches zero even faster than a Gaussian distribution of width $\sigma$. This implies that if $p$-values based on a Gaussian are used, the test is slightly conservative, and will not give extra false positives.

### 3.5. Statistical tests of normality

An image of a uniform underlying intensity with Rician noise has a spatially stationary noise distribution. The distribution of the difference between two such images is symmetric.

To test whether this distribution is close to Gaussian, the Kolmogorov-Smirnov (KS) test was employed as follows. We created two images of a uniform intensity $A$ with Rician distributed noise, and computed the difference between the noisy images. The KS test was applied to the difference image. The null hypothesis of the KS test is that the data are normally distributed, and this is rejected if the $p$-value of the KS test statistic is below 0.05. For a number of intensities $A$, images of different sizes were tested, and for each size and intensity, the test was repeated 32 times. Table 2 shows the mean $p$-values of the KS test statistics for each size, with intensity $A = 1$ and $A = 5$. As a reference, 32 images of the same size containing $N(0, 1)$ noise were also tested, and their mean $p$-values are in the right column. This table shows that deviations from normality can only be detected in very large images with low intensities: for high intensities, they are too small to measure.

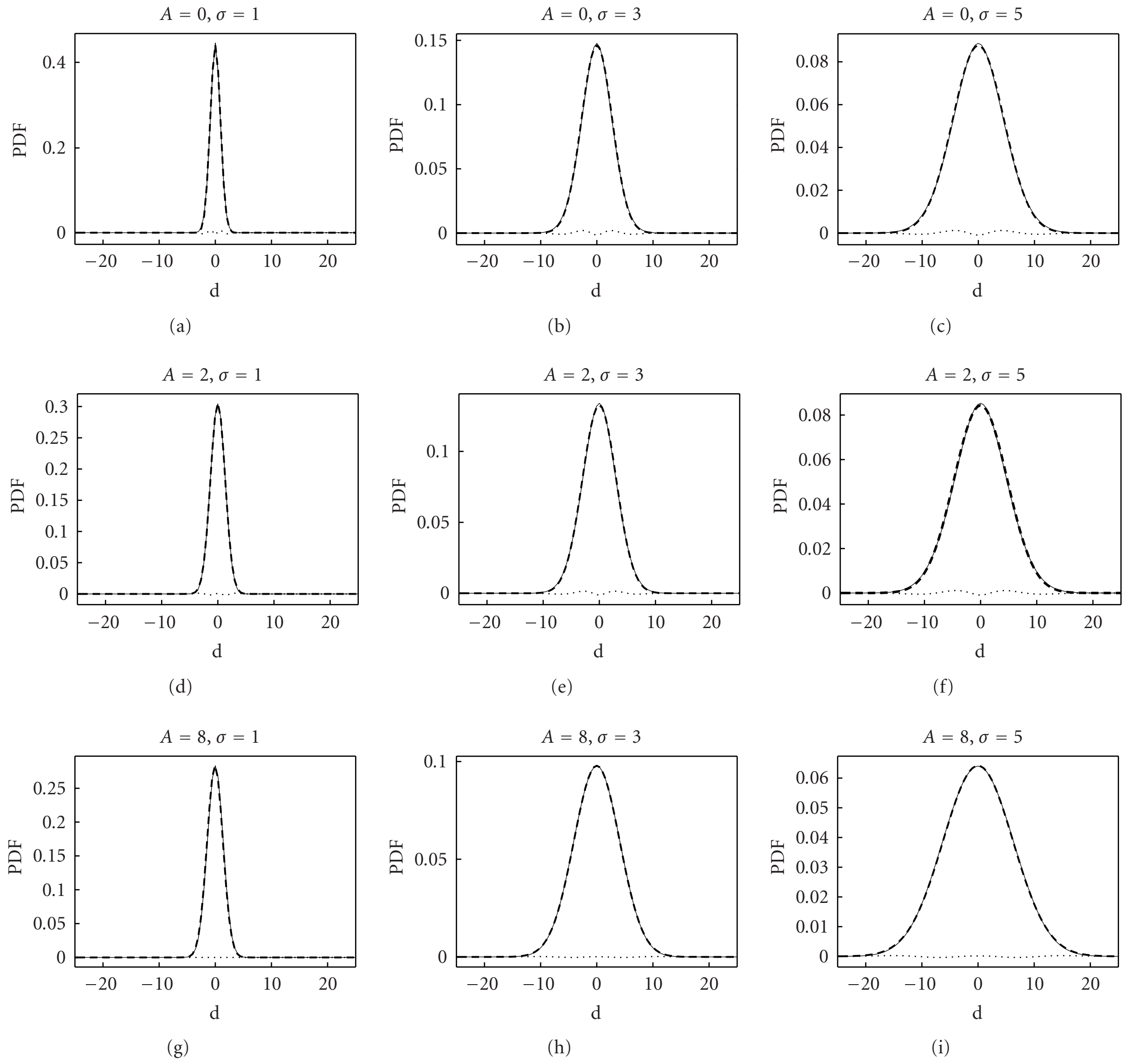

FIGURE 2: The exact null distribution $C_{A,\sigma}(s)$ (solid), the fitted Gaussian (dashed), and the difference between $C_{A,\sigma}(s)$ and the Gaussian (dotted). Note that the fitted Gaussian is hardly distinguishable from the exact distribution.

### 3.6. *Parameter estimation in fMRI with the general linear model*

For fMRI analysis, the possibility of accurately estimating the parameters of the noise is at least as important as using the right noise model. We tested the applicability of the GLM (see Section 3.1) by estimating the noise parameters in difference images created in the same way as $s(\mathbf{x})$ in Section 3.2, and comparing them with the real underlying parameters.

A matrix $\mathbf{e}$ of error signals was created by making a time series of 128 difference images. The standard deviation of the temporal noise was computed in each voxel. Table 3 shows, for the same input $A$ and $\sigma$ as before, the measured temporal standard deviation $\sigma^{\text{temp}}$, the mean standard deviation $\sigma_s$ in the difference images (which equals $\sqrt{2}\sigma_r$, see Section 3.2), and the ratio $\sigma_s/\sigma^{\text{temp}}$. It shows that the standard deviation $\sigma_s$ is very accurately predicted by formula (11).

### 3.7. *Evaluation of the test results*

The statistical tests, the analytical results, and the numerical computations, all show that the difference between two MR images whose intensities are Rician distributed, can be very well approximated by a Gaussian distribution. The approximation is closest for high SNR, but is still very good for lower SNR. Given the parameters $A$ and $\sigma$ of the Rician spatial noise in a series of MR images and defining null images as pairwise difference images, the parameters of the Gaussian distribution that describes the temporal noise can be accurately estimated.

Table 2: $p$-values produced by the KS test for the difference between images with Rician distributed noise with signal amplitudes $A = 1$ and $A = 5$, and for images of the same size with $N(0, 1)$-noise.

| Size | $p$-value ($A = 1$) | $p$-value ($A = 5$) | $p$-value $N(0, 1)$ |
|---|---|---|---|
| $2 \times 2$ | 0.6573 | 0.5607 | 0.4569 |
| $4 \times 4$ | 0.5761 | 0.5565 | 0.4249 |
| $8 \times 8$ | 0.5511 | 0.5493 | 0.4894 |
| $16 \times 16$ | 0.5801 | 0.5564 | 0.5854 |
| $32 \times 32$ | 0.5833 | 0.5378 | 0.5946 |
| $64 \times 64$ | 0.5629 | 0.4869 | 0.4816 |
| $128 \times 128$ | 0.5270 | 0.5426 | 0.5147 |
| $256 \times 256$ | 0.4390 | 0.5554 | 0.5225 |
| $512 \times 512$ | 0.3210 | 0.5219 | 0.4006 |
| $1024 \times 1024$ | 0.0587 | 0.5236 | 0.5037 |

Table 3: Measured temporal standard deviation $\sigma^{\text{temp}}$, the mean standard deviation $\sigma_s$ in the difference images, and the ratio $\sigma_s/\sigma^{\text{temp}}$.

| $A$ | $\sigma$ | $\sigma_s$ | $\sigma^{\text{temp}}$ | $\sigma_s/\sigma^{\text{temp}}$ |
|---|---|---|---|---|
| 0 | 1 | 1.4280 | 1.4338 | 0.9959 |
| 0 | 3 | 4.2854 | 4.3017 | 0.9962 |
| 0 | 5 | 7.1352 | 7.1667 | 0.9956 |
| 2 | 1 | 1.8001 | 1.8071 | 0.9961 |
| 2 | 3 | 4.4708 | 4.4890 | 0.9959 |
| 2 | 5 | 7.2472 | 7.2815 | 0.9953 |
| 8 | 1 | 2.1579 | 2.1667 | 0.9959 |
| 8 | 3 | 5.7924 | 5.8157 | 0.9960 |
| 8 | 5 | 8.5195 | 8.5542 | 0.9959 |

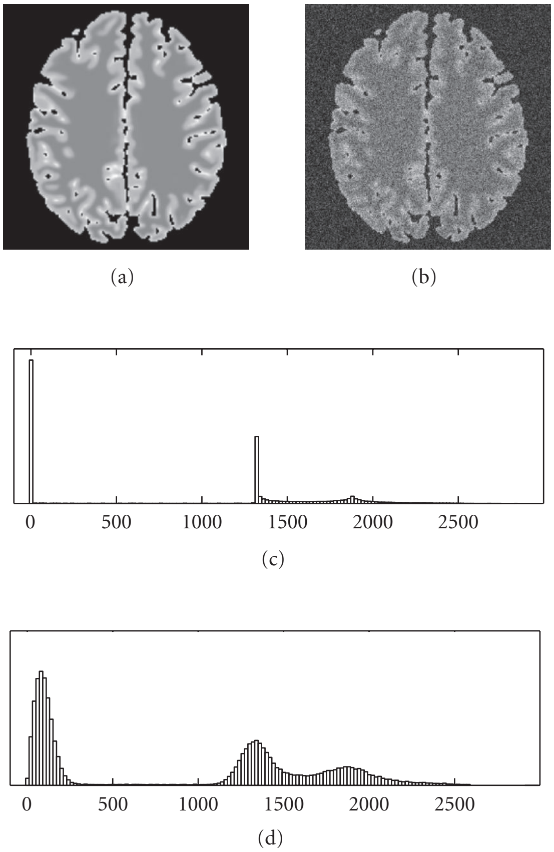

Figure 3: (a) A noise-free T2-weighted MR image. (b) Image (a) with Rician noise of $\sigma = 81.67$ (SNR 10 dB). The histogram of a noise-free T2*-weighted MR image (c) and of the same image with Rician noise of $\sigma = 81.67$, SNR = 10 dB (d).

### 4. THE NOISE DISTRIBUTION IN fMRI

Images in an fMRI time series have a range of intensities, so the noise distribution is a sum of Rician PDFs (sums of Gamma PDFs have also been used, see [15] for an example). For each intensity $A$ in the image, the noise is distributed differently (see Figure 3(c)), and this will have an influence on the parameter estimates of the GLM. Areas with a "true" grey value of 0, like the space around the body, have Rayleigh-distributed noise, and the areas with higher grey values have more symmetric distributions, which are quite similar to a Gaussian, and they are centered around the grey value at that location. The total noise distribution is a mixture of all those distributions. The question is whether the conclusions about the noise in the difference image obtained in Section 2 also hold for noisy images with mixed distributions.

### 4.1. Shape of the noise distribution in MR images

A simulated MR image was acquired from the BrainWeb Magnetic Resonance Imaging simulator [20] with the following parameters: modality T2, slice thickness 1 mm, noise 0%, intensity nonuniformity 0%. Nonbrain voxels were excluded with the Brain Extraction Tool [21]. This noise-free T2*-weighted image (Figure 3(a)) was contaminated by Rician noise with a known $\sigma$ (see Figure 3(b)). A residual image was obtained by subtracting the original MR image from the noisy MR image, and a null image was made using the procedure proposed in Section 3, that is, as the difference between two MR images containing Rician noise.

The dissimilarity between a Rician distribution and a Gaussian is largest for low signal intensities $A$. The previous section showed that the difference between two images of a uniform intensity $A$ and Rician noise has zero mean and is near-Gaussian distributed, also for low signal intensities. This section examines the difference images when the noise-free images contain more than one intensity. Figure 4 shows the histograms of a noisy MR image, the difference between a noisy MR image and the noise-free image, and the difference between two noisy MR images, respectively. The histograms were computed for a range of values for $\sigma$ and are presented together as surface plots. As $\sigma$ decreases, the histogram of the noisy MR image changes from one Rayleigh-like PDF to a number of near-Gaussian PDFs (see Figure 4(a)). The histogram of the noisy image after subtraction of the original is asymmetric for high $\sigma$, and becomes more symmetric as $\sigma$ decreases (see Figure 4(b)). The histogram of the difference images is symmetric for all $\sigma$ (see Figure 4(c)).

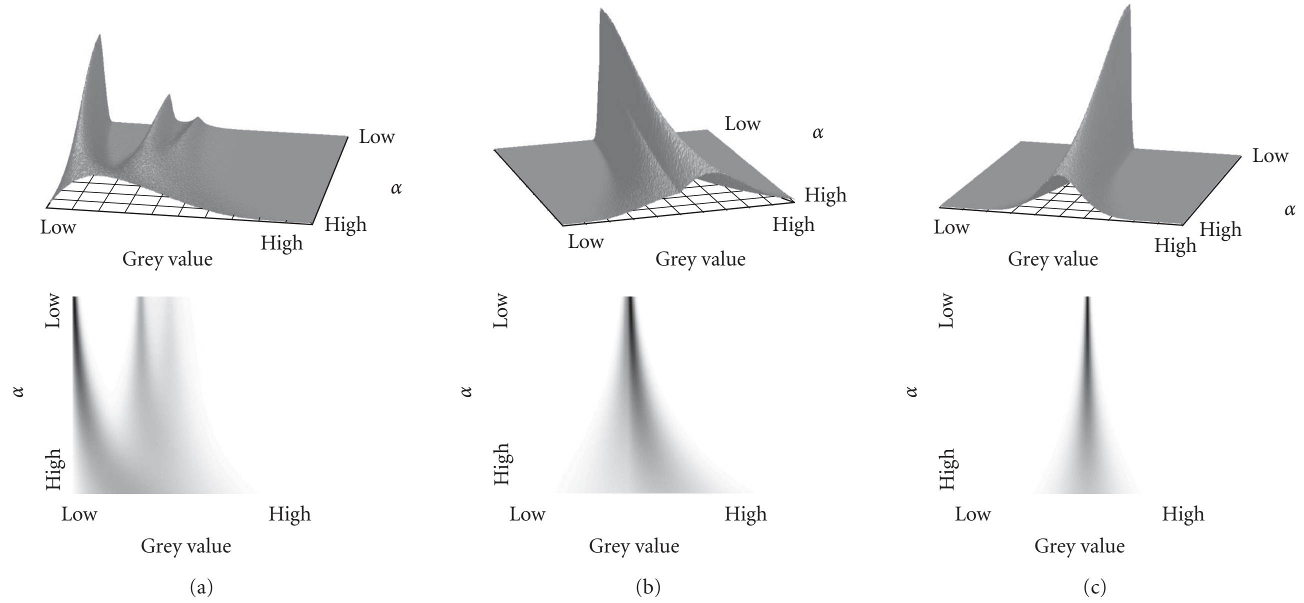

Figure 4: (a) Histogram of a noisy MR image, (b) histogram of the difference between a noisy MR image and the noise-free MR image, and (c) histogram of the difference of two noisy MR images, for various $\sigma$. Top: surface plots, bottom: grey-value maps.

### 4.2. *Time series of MR images*

A time series of 164 EPI scans was made on a 3 Tesla Intera scanner (Philips Medical Systems, The Netherlands), with repetition time TR = 3 seconds, volume size = $64 \times 64 \times 46$ voxels of $3.5 \times 3.5 \times 3.5\ \text{mm}^3$. No stimuli were presented, and the null hypothesis of no activation was assumed to be true throughout the experiment. Alignment of the images was done with SPM′99 program [13].

The time series was split in two disjoint sets: $TS_1$ (images $1, \ldots, 82$) and $TS_2$ (images $83, \ldots, 164$). The noise of $TS_1$ was centered around 0 by subtracting the time series mean image of $TS_1$ from each image. Note that although this is a common procedure in fMRI analysis, this means treating Rician noise as additive noise. To obtain an image with a symmetric noise distribution, difference images were made by subtracting the corresponding image of $TS_2$ from each image of $TS_1$.

The histogram of the time series mean image (see Figure 5) was used to divide the images into three intensity ranges: low intensity (grey value $0, \ldots, 300$), medium intensity (grey value $301, \ldots, 600$), and high intensity (grey value $> 600$).

Figure 6 shows the histograms of the grey values in the resulting time series within the three ranges. Gaussians were fitted to the histograms with the Levenberg-Marquardt curve-fitting algorithm. For medium and high intensities, the time series histograms show no significant asymmetries. For low intensities however, the time series $TS_1$ after subtracting the mean has an asymmetric histogram, while the time series $TS_1$ after subtracting $TS_2$ has a symmetric histogram. The fits are never perfect, except in the case of low intensities and after subtracting $TS_2$. In that case, the intensity distribution has one predominant intensity ($A = 0$, see Figure 5), and the difference distribution is close to those in the $A = 0$ cases of the previous section. In the other cases, the noise originates from voxels with various intensities, and the noise distribution resembles a mixture of Gaussians with mean $\mu = 0$ and various $\sigma$.

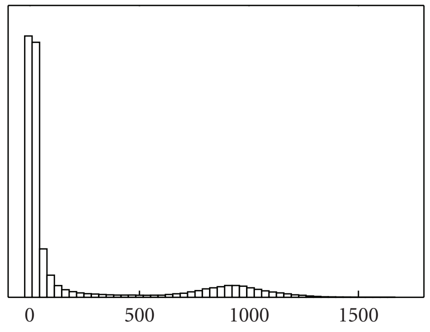

Figure 5: Histogram of the time series mean image of $TS_1$.

Because the amount of asymmetry in the medium and high grey-value ranges is very small, the combination of thresholding and subtracting the time series mean may solve most of the problems concerning the Rician distribution of the noise. However, the new method presented in this paper of subtracting a second-time series is preferable: it has proved to yield symmetric noise distributions in all measurements considered.

### 4.3. *Implications for fMRI designs*

The assumption of Gaussian noise in the analysis of fMRI data should be used with care. Relying on the robustness of

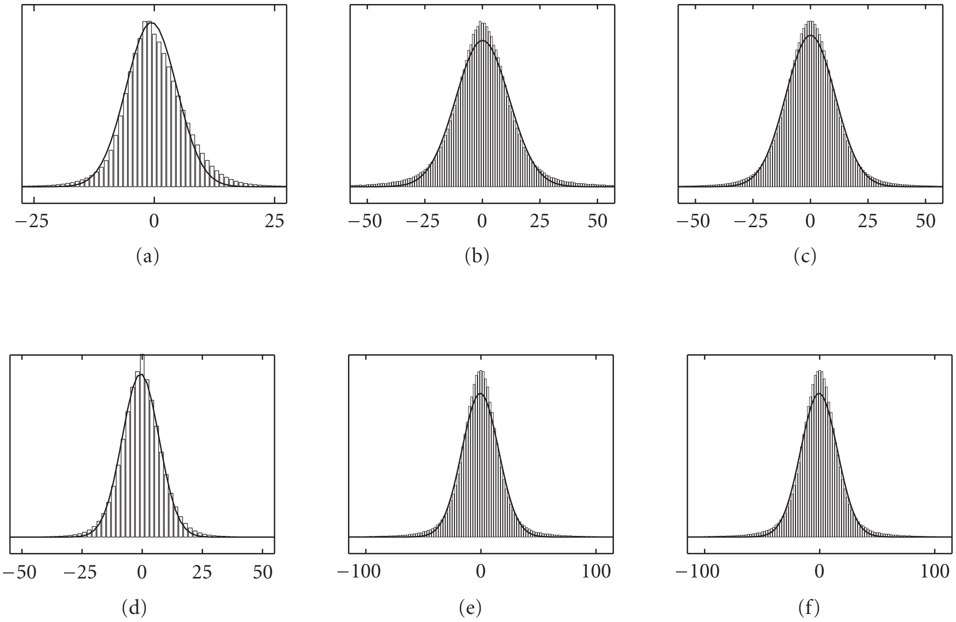

FIGURE 6: Histograms of three intensity ranges of the images in the time series. Top: time series $TS_1$ after subtracting the time series mean: (a) low intensity, (b) medium intensity, (c) high intensity. Bottom: time series $TS_1$ after subtracting the corresponding images of time series $TS_2$: (d) low intensity, (e) medium intensity, (f) high intensity.

the standard tests most often works, but it does not solve the problem of the asymmetric noise distribution. A recent maximum-likelihood test based on the Rician distribution shows to be as powerful as the GLM-based test with a high SNR, but performs much better with a low SNR [22]. For using the assumption of Gaussian noise, difference distributions like the one presented here will be required. The example presented here of using an extra data set for every experiment is difficult for large studies, but this can be solved in a more practical way: a relatively small set of "null data" can be reused after randomisation in the time dimension. The only change in the formula for the GLM (7) is using $\mathbf{Y} - \mathbf{Y}_0$ instead of $\mathbf{Y}$, with $\mathbf{Y}_0$ the resting-state data set. It is trivial to see that this does not change the way the estimates are computed, even if different (more complex) design matrices $\mathbf{X}$ are used.

### 5. CONCLUSIONS

We have presented a noise model in BOLD fMRI that takes into account the Rician distribution of MR noise known from the literature. BOLD noise was defined as the difference between two MR images with Rician noise. We investigated the properties of the difference image under the null hypothesis (no brain activation), which is needed to determine $p$-values in a statistical analysis. The problem was studied in several complementary ways: analytical calculation, numerical simulation, statistical estimation, and experimental validation on real EPI data. An analytic expression was derived for the statistical null distribution $C_{A,\sigma}(s)$ as an integral in terms of two underlying Rician probability densities with parameters $A$ and $\sigma$. From this basic formula, analytical expressions were derived for the mean and standard deviation of the null distribution, as well as for its tail, that is, its asymptotic behaviour as $s$ goes to infinity.

The null distribution $C_{A,\sigma}(s)$ was numerically approximated by a Gaussian function with the Levenberg-Marquardt nonlinear curve-fitting algorithm. The approximation by a Gaussian distribution was very good, with the accuracy increasing with SNR (i.e., $A/\sigma$). The standard deviation of the fitted Gaussian was found to be in excellent agreement with the exact standard deviation $\sigma_s$ derived from the analytical expressions.

The statistical properties of the noise were examined in two ways. The Kolmogorov-Smirnov test was applied to difference images of noise-only images with Rician distributed noise. A second test using the general linear model (GLM) compared the estimated noise parameters to the value predicted by the model, and showed that the agreement is excellent.

From the analytical results, the numerical computations, and the statistical tests, we concluded that the assumption of Gaussian distributed noise used in the fMRI literature could be justified. That is, the difference between two images whose intensities follow a Rice distribution can be very well approximated by a Gaussian distribution. The approximation is closest for high SNR, but is still quite good for lower SNR. Given the parameters $A$ and $\sigma$ of the Rician spatial noise in a series of MR images, the standard deviation of the Gaussian that describes the temporal noise can be accurately predicted.

The noise model was tested on simulated and real MR images. In a test that contaminated noise-free MR templates with Rician noise, MR noise was shown to have an asymmetric distribution when it is—incorrectly—treated as additive noise. As in the test with noise-only images, difference images of noisy MR pictures were found to have a symmetric distribution. The consequence for fMRI time series analysis is that subtracting the time series mean does not get rid of the asymmetry in temporal noise.

We tested thresholding the MR images as a fast and simple alternative to the difference image approach: it can remove asymmetry in the noise distribution to a large extent, depending on the robustness of the test that is used. Subtracting a second time series from the time series being analysed yields symmetric and close to Gaussian distributed noise.

### APPENDIX

### A. MATHEMATICAL ANALYSIS OF THE NULL DISTRIBUTION

This appendix presents the derivations of the exact analytical results in Section 3 on the distribution of the difference signal under the null hypothesis. Extensive use is made of the concept of asymptotic expansions. We first provide a few formal definitions. Let $\phi(x)$ and $\psi(x)$ be two functions defined for $x \geq x_0$. One writes $\phi(x) = O(\psi(x))$, $x \to \infty$, when constants $K$ and $x_1$ exist such that $|\phi(x)| \leq K|\psi(x)|$ for $x \geq x_1$. We call $\sum_{n=0}^{\infty} a_n\phi_n(x)$ the asymptotic expansion of a function $f$ when, for every $N$, $|f(x) - \sum_{n=0}^{N} a_n\phi_n(x)| = O(\phi_{N+1}(x))$, and write

$$f(x) \sim \sum_{n=0}^{\infty} a_n\phi_n(x), \quad x \longrightarrow \infty. \tag{A.1}$$

Below, we only use the first term in the asymptotic expansion of some special functions (error function, Bessel function), and use the shorthand notation

$$f(x) \sim a_0\phi_0(x), \quad x \longrightarrow \infty. \tag{A.2}$$

To make this precise, one has to refer to the full asymptotic expansions, as can be found in Abramowitz and Stegun [19]; for easy reference, we refer to the relevant sections of this handbook at the appropriate places.

### A.1. Mean and variance of the null distribution

First, the mean $\mu_s$ is zero because of the symmetry of $C_{A,\sigma}(s)$. Second, since the mean is zero, the variance of the null distribution satisfies (see (10))

$$\begin{aligned}\sigma_s^2 &= \int_{-\infty}^{\infty} ds\, s^2 C(s)\\ &= \int_{-\infty}^{\infty} ds\, s^2 \int_0^{\infty} dr_1 \int_0^{\infty} dr_2\, p_{A,\sigma}(r_1)p_{A,\sigma}(r_2)\delta(r_2 - r_1 - |s|)\\ &= \int_0^{\infty} dr_1 \int_0^{\infty} dr_2\, p_{A,\sigma}(r_1)p_{A,\sigma}(r_2)\int_{-\infty}^{\infty} ds s^2\delta(r_2 - r_1 - |s|),\end{aligned} \tag{A.3}$$

where $\delta(\cdot)$ denotes the Dirac delta function. Since $\delta(r_2 - r_1 - |s|)$ is zero except when $r_2 - r_1 - |s| = 0$, we find

$$\begin{aligned}\sigma_s^2 &= \int_0^{\infty} dr_1 \int_0^{\infty} dr_2\, p_{A,\sigma}(r_1)p_{A,\sigma}(r_2)(r_1 - r_2)^2\\ &= \int_0^{\infty} dr_1 \int_0^{\infty} dr_2\, p_{A,\sigma}(r_1)p_{A,\sigma}(r_2)(r_1^2 + r_2^2 - 2r_1r_2)\\ &= \int_0^{\infty} dr_1 r_1^2 p_{A,\sigma}(r_1) + \int_0^{\infty} dr_2 r_2^2 p_{A,\sigma}(r_2)\\ &\quad - 2\Big(\int_0^{\infty} dr_1 r_1 p_{A,\sigma}(r_1)\Big)\Big(\int_0^{\infty} dr_2 r_2 p_{A,\sigma}(r_2)\Big)\\ &= 2\mathbb{E}(r^2) - 2\mathbb{E}(r)^2 = 2\sigma_r^2.\end{aligned} \tag{A.4}$$

Here $\mathbb{E}(\cdots)$ denotes the average of the quantity within the brackets. So we have found that $\sigma_s^2 = 2\sigma_r^2$, which directly yields (11).

### A.2. Exact form of the null distribution in the Rayleigh case

Substituting the form (12) of the Rayleigh distribution in expression (10), we find

$$C_{0,\sigma}(s) = \int_0^{\infty} dr \frac{r}{\sigma^2} e^{-r^2/2\sigma^2} \frac{r + |s|}{\sigma^2} e^{-(r+s)^2/2\sigma^2}. \tag{A.5}$$

Putting $r/\sigma = x$, $|s|/\sigma = q$, $A/\sigma = a$, we find after some algebra

$$C_{0,\sigma}(s) = \frac{1}{\sigma}\int_0^{\infty} dx \Big\{\Big(x + \frac{q}{2}\Big)^2 - \frac{q^2}{4}\Big\} e^{-(x+q/2)^2 - q^2/4}. \tag{A.6}$$

Again, putting $y = x + q/2$,

$$C_{0,\sigma}(s) = \frac{1}{\sigma} e^{-q^2/4}\int_{q/2}^{\infty} dy \Big(y^2 - \frac{q^2}{4}\Big) e^{-y^2}. \tag{A.7}$$

Writing $\tau = q/2$, we can write this integral as the sum of two terms, each of which can be expressed in terms of the complementary error function

$$C_{0,\sigma}(s) = \frac{1}{\sigma} e^{-\tau^2} S_2 - \frac{1}{\sigma} e^{-\tau^2}\tau^2 S_0, \tag{A.8}$$

where

$$\begin{aligned} S_0 &= \int_{\tau}^{\infty} dy e^{-y^2} = \frac{\sqrt{\pi}}{2}\operatorname{erfc}(\tau),\\ S_2 &= \int_{\tau}^{\infty} dy y^2 e^{-y^2} = \frac{1}{2}\tau e^{-\tau^2} + \frac{\sqrt{\pi}}{4}\operatorname{erfc}(\tau).\end{aligned} \tag{A.9}$$

Substitution of these expressions in (A.8) yields

$$C_{0,\sigma}(s) = \frac{1}{2\sigma} e^{-\tau^2}\Big\{\tau e^{-\tau^2} + \frac{\sqrt{\pi}}{2}(1 - 2\tau^2)\operatorname{erfc}(\tau)\Big\}. \tag{A.10}$$

Reexpressing $\tau$ in terms of the original variable $s$ (i.e., $\tau = q/2 = |s|/(2\sigma)$), we obtain formula (13).

### A.3. Tails of the null distribution

We consider the limiting case of low versus high SNR, that is, $A = 0$ and $A/\sigma$ large.

$A = 0$. This is the Rayleigh case, for which we have derived an exact expression for the null distribution, see formula (13). When $s$ is large, we can use the asymptotic behaviour of the error function [19, section 7.1.23]

$$\operatorname{erfc}(z) \sim \frac{1}{\sqrt{\pi} z} e^{-z^2}, \quad z \longrightarrow \infty. \tag{A.11}$$

Substituting this in (13), we find (after rearrangement of terms)

$$C_{0,\sigma}(s) \sim \frac{1}{2|s|} e^{-s^2/2\sigma^2}, \quad s \longrightarrow \infty, \tag{A.12}$$

which behaves as a Gaussian tail of width $\sigma$ multiplied by a factor $1/|s|$.

*$A/\sigma$ large*. Since $A/\sigma$ is large, we apply the Gaussian approximation of the Rice distribution:

$$p_{A,\sigma}(r) \sim \frac{1}{\sqrt{2\pi\sigma^2}} e^{-(r-A)^2/2\sigma^2}. \tag{A.13}$$

As shown in [3], this approximation is already accurate for $A \geq 2\sigma$. This formula is easy to derive by using the asymptotic expansion of the Bessel function $I_0$ as given in [19, Section 9.7.1]. Substituting this in (10), we get

$$C_{A,\sigma}(s) \sim \int_0^\infty \mathrm{d}r \frac{1}{2\pi\sigma^2} e^{-(r-A)^2/2\sigma^2} e^{-(r+|s|-A)^2/2\sigma^2}. \tag{A.14}$$

Putting $r/\sigma = x$, $|s|/\sigma = q$, $A/\sigma = a$, we find after some algebra

$$\begin{aligned} C_{A,\sigma}(s) &\sim \frac{1}{2\pi\sigma} \int_0^\infty \mathrm{d}x e^{-(x-a)^2/2} e^{-(x+q-a)^2/2} \\ &= \frac{1}{2\pi\sigma} e^{-q^2/4} \int_0^\infty \mathrm{d}x e^{-(x+q/2-a)^2}. \end{aligned} \tag{A.15}$$

Again, putting $y = x + q/2 - a$,

$$\begin{aligned} C_{A,\sigma}(s) &\sim \frac{1}{2\pi\sigma} e^{-q^2/4} \int_{q/2-a}^\infty \mathrm{d}y e^{-y^2} \\ &= \frac{1}{2\pi\sigma} e^{-q^2/4} \frac{\sqrt{\pi}}{2} \operatorname{erfc}\left(\frac{q}{2} - a\right). \end{aligned} \tag{A.16}$$

In terms of the original variable $s$,

$$C_{A,\sigma}(s) \sim \frac{1}{4\sqrt{\pi}\sigma} e^{-s^2/4\sigma^2} \operatorname{erfc}\left(\frac{|s|/2 - A}{\sigma}\right). \tag{A.17}$$

Applying the asymptotic expansion (A.11) of the erfc function for large argument, we find

$$C_{A,\sigma}(s) \sim \frac{1}{2\pi(|s| - 2A)} e^{-((|s|-A)^2+A^2)/2\sigma^2}. \tag{A.18}$$

Finally, since $|s|$ is large, we can replace $|s| - 2A$ by $|s|$:

$$C_{A,\sigma}(s) \sim \text{constant} \cdot \frac{1}{|s|} e^{-(|s|-A)^2/2\sigma^2}, \quad s \longrightarrow \infty, \tag{A.19}$$

which again behaves as a Gaussian tail of width $\sigma$ multiplied by a factor $1/|s|$.

### ACKNOWLEDGMENTS

This research was part of the project “wavelets and their applications,” funded by the Dutch National Science Foundation (NWO), Project no. 613.006.570. We thank Dr. N. M. Temme of the Centre for Mathematics and Computer Science in Amsterdam for helpful comments on the asymptotic expansions used in this paper.

### REFERENCES

[1] W. A. Edelstein, P. A. Bottomley, and L. M. Pfeifer, “A signal-to-noise calibration procedure for NMR imaging systems,” *Medical Physics*, vol. 11, no. 2, pp. 180–185, 1984.

[2] R. M. Henkelman, “Measurement of signal intensities in the presence of noise in MR images,” *Medical Physics*, vol. 12, no. 2, pp. 232–233, 1985.

[3] H. Gudbjartsson and S. Patz, “The Rician distribution of noisy MRI data,” *Magnetic Resonance in Medicine*, vol. 34, no. 6, pp. 910–914, 1995.

[4] J. Sijbers, A. J. den Dekker, P. Scheunders, and D. Van Dyck, “Maximum-likelihood estimation of Rician distribution parameters,” *IEEE Transactions on Medical Imaging*, vol. 17, no. 3, pp. 357–361, 1998.

[5] R. D. Nowak, “Wavelet-based Rician noise removal for magnetic resonance imaging,” *IEEE Transactions on Image Processing*, vol. 8, no. 10, pp. 1408–1419, 1999.

[6] J. C. Wood and K. M. Johnson, “Wavelet packet denoising of magnetic resonance images: importance of Rician noise at low SNR,” *Magnetic Resonance in Medicine*, vol. 41, no. 3, pp. 631–635, 1999.

[7] A. Pižurica, W. Philips, I. Lemahieu, and M. Acheroy, “A versatile wavelet domain noise filtration technique for medical imaging,” *IEEE Transactions on Medical Imaging*, vol. 22, no. 3, pp. 323–331, 2003.

[8] S. O. Rice, “Mathematical analysis of random noise,” *The Bell System Technical Journal*, vol. 24, no. 1, pp. 46–156, 1945.

[9] G. K. Aguirre, E. Zarahn, and M. D’Esposito, “Empirical analyses of BOLD fMRI statistics. II. Spatially smoothed data collected under null-hypothesis and experimental conditions,” *NeuroImage*, vol. 5, no. 3, pp. 199–212, 1997.

[10] K. J. Friston, O. Josephs, E. Zarahn, A. P. Holmes, S. Rouquette, and J.-B. Poline, “To smooth or not to smooth? Bias and efficiency in fMRI time-series analysis,” *NeuroImage*, vol. 12, no. 2, pp. 196–208, 2000.

[11] M. J. Fadili and E. T. Bullmore, “Wavelet-generalized least squares: a new BLU estimator of linear regression models with 1/f errors,” *NeuroImage*, vol. 15, no. 1, pp. 217–232, 2002.

[12] K. J. Friston, C. D. Frith, R. Turner, and R. S. J. Frackowiak, "Characterizing evoked hemodynamics with fMRI," *NeuroImage*, vol. 2, no. 2 Pt 1, pp. 157–165, 1995.

[13] K. J. Friston, A. P. Holmes, K. J. Worsley, J.-P. Poline, C. D. Frith, and R. S. J. Frackowiak, "Statistical parametric maps in functional imaging: a general linear approach," *Human Brain Mapping*, vol. 2, no. 4, pp. 189–210, 1994, http://www.fil.ion.ucl.ac.uk/spm/.

[14] K. J. Worsley and K. J. Friston, "Analysis of fMRI time-series revisited - again," *NeuroImage*, vol. 2, no. 3, pp. 173–181, 1995.

[15] S. J. Hanson and B. M. Bly, "The distribution of BOLD susceptibility effects in the brain is non-Gaussian," *NeuroReport*, vol. 12, no. 9, pp. 1971–1977, 2001.

[16] W.-L. Luo and T. E. Nichols, "Diagnosis and exploration of massively univariate neuroimaging models," *NeuroImage*, vol. 19, no. 3, pp. 1014–1032, 2003.

[17] K. M. Petersson, T. E. Nichols, J.-B. Poline, and A. P. Holmes, "Statistical limitations in functional neuroimaging II. Signal detection and statistical inference," *Philosophical Transactions of the Royal Society*, vol. 354, no. 1387, pp. 1261–1281, 1999.

[18] J. Sijbers, A. J. den Dekker, J. Van Audekerke, M. Verhoye, and D. Van Dyck, "Estimation of the noise in magnitude MR images," *Magnetic Resonance Imaging*, vol. 16, no. 1, pp. 87–90, 1998.

[19] M. Abramowitz and I. A. Stegun, Eds., *Handbook of Mathematical Functions*, vol. 55 of *Applied Mathematics Series*, National Bureau of Standards USA, Washington, DC, USA, 10th edition, 1972.

[20] R. K.-S. Kwan, A. C. Evans, and G. B. Pike, "An extensible MRI simulator for post-processing evaluation," in *Proceedings of 4th International Conference Visualization in Biomedical Computing (VBC '96)*, vol. 1131 of *Lecture Notes in Computer Science*, pp. 135–140, Hamburg, Germany, September 1996, http://www.bic.mni.mcgill.ca/brainweb.

[21] S. M. Smith, "Fast robust automated brain extraction," *Human Brain Mapping*, vol. 17, no. 3, pp. 143–155, 2002.

[22] A. J. den Dekker and J. Sijbers, "Implications of the Rician distribution for fMRI generalized likelihood ratio tests," *Magnetic Resonance Imaging*, vol. 23, no. 9, pp. 953–959, 2005.

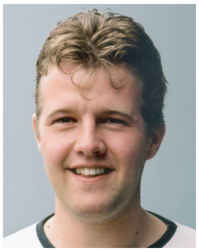

**Alle Meije Wink** received the M.S. degree (1999) in computing science from the University of Twente, The Netherlands. He has worked in image analysis (1999) at the University Hospital Groningen, The Netherlands, and received a Ph.D. degree (2004) in computing science, with a focus on fMRI analysis, from the University of Groningen. He is currently a Research Associate in the Brain Mapping Unit, University of Cambridge, United Kingdom.

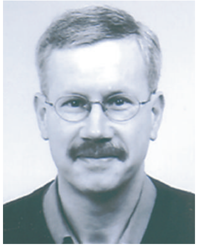

**Jos B. T. M. Roerdink** received his M.S. degree (1979) in theoretical physics from the University of Nijmegen, the Netherlands. Following his Ph.D. degree (1983) from the University of Utrecht and a two-year position (1983–1985) as a Postdoctoral Fellow at the University of California, San Diego, both in the area of stochastic processes, he joined the Centre for Mathematics and Computer Science in Amsterdam. There he worked from 1986 to 1992 on image processing and tomographic reconstruction. He was appointed as an Associate Professor (1992) and Full Professor (2003), respectively, at the Institute for Mathematics and Computing Science of the University of Groningen, where he currently holds a Chair in scientific visualization and computer graphics. His research interests include mathematical morphology, biomedical visualization, neuroimaging, and bioinformatics.

